# Brazilian guidelines in critical care: let's face this challenge...

**DOI:** 10.5935/0103-507X.20160046

**Published:** 2016

**Authors:** Thiago Costa Lisboa, Alexandre Biasi Cavalcanti, Suzana Margareth Ajeje Lobo

**Affiliations:** 1 Rede Institucional de Pesquisa e Inovação em Medicina Intensiva (RIPIMI), Complexo Hospitalar Santa Casa - Porto Alegre (RS), Brazil.; 2 Critical Care Department and Infection Control Committee, Hospital de Clinicas de Porto Alegre - Porto Alegre (RS), Brazil.; 3 Research Institute, Hospital do Coração - São Paulo (SP), Brazil.; 4 Faculdade de Medicina de São José do Rio Preto - São José do Rio Preto (SP), Brazil.

Clinical practice guidelines are useful tools to improve delivery of the best care, based on the best available evidence, for our patients. They help practitioners to make clinical decisions and might help to ensure the proper allocation of resources in public health policy.^(1)^ Therefore, health care guidelines and their appropriate implementation are of interest to national organizations, professional societies, health care providers, policy-makers, patients, and the public.^(2)^

Guidelines must be grounded on the evidence-based medicine paradigm, which is comprised of four fundamental aspects: recognition of the patient's problem and the construction of a structured clinical question, effective and extensive search of the medical literature to obtain the best available evidence, critical appraisal of the evidence, and, finally, integration of the evidence in patient decision making to determine the best clinical care for the patient.^(3)^

The *Associação de Medicina Intensiva Brasileira* (AMIB) has settled on a project called "*Diretrizes AMIB*" to develop evidence-based local medicine guidelines in several areas of critical care. In this issue of our Journal, the first clinical guidelines resulting from this initiative are published. AMIB and the *Associação Brasileira de Transplante de* Órgãos (ABTO) have developed guidelines for the recognition, evaluation, and validation of potential donors for organ transplantation. This is a crucial aspect, as organ transplantation is the last resource available for several health conditions and availability of organs is limited and expensive, with high costs associated. Therefore, these guidelines aim to improve our ability to recognize and diagnose brain death and to assess eligibility for organ donation.^(4)^

The authors divide the guidelines into four subgroups: screening of potential donors; brain death diagnosis; criteria for the selection of potential donor; and organ-specific contraindications. The authors defined the strength of recommendation according to the GRADE methodology (Grading of Recommendation, Assessment, Development, and Evaluation)^(5)^ and the level of evidence according to the *Projeto Diretrizes* da *Associação Médica Brasileira* (AMB) e do *Conselho Federal de Medicina* (CFM).^(6)^ The authors then built a set of recommendations aiming to establish more protocolled care of the potential donor, as well as identifying and selecting patients, and optimizing the use of resources. The authors made a substantial effort to summarize the best evidence available, especially in a subject such as this, in which few high quality studies are published. Certainly, this project will provide guidance on the most effective management methods to screen, identify, and select a donor in Brazil, thus improving organ availability and transplantation.

These guidelines were evaluated by members of the editorial board of the Journal who considered qualitative aspects of its methodology. Every clinical recommendation and guideline should be assessed to ensure that potential biases of guideline development have been adequately addressed and that the recommendations are both internally and externally valid and are feasible for practice. This is a complex process, and some tools are available in the literature (e.g., AGREE II). The assessment includes judgments about the methods used for developing the guidelines, the components of the final recommendations, and the factors that are linked to their uptake.^(1)^ Based on AGREE II, any guideline should be evaluated according to six domains, and this methodology will be used during the whole process in the project *Diretrizes AMIB*.

The first domain refers to the "Scope and purpose" of the guideline. Here, reviewers will evaluate whether objectives, research questions, and study populations are well defined and declare the expected health benefit specific to the clinical problem or health topic. Then, the second domain assesses "Stakeholder involvement." The guideline will be assessed regarding authors' involvement in the process and to ensure that all groups potentially interested in the results are represented and that targets are well defined. Undeclared conflicts of interest may be discovered when assessing these points and should be considered in guideline quality analysis as along with editorial independence. The next domain is "Rigor of development," in which all methodologies of guideline building will be evaluated. The domain will address search criteria, how evidence was selected and graded, the strength of recommendations and how were they stated, and how well these aspects were described. Next, the fourth domain will assess "Clarity of presentation." This refers to how specific and unambiguous the recommendations provided are, what options for management are offered, and how to handle uncertainty about the best care option when evidence is not definite. The fifth domain is "Applicability." A proper guideline should describe facilitators and barriers to its implementation as well as discuss resources and tools for monitoring or auditing its implementation. The last domain refers to "Editorial independence" and aims to assure that the views of the funding body have not influenced the guideline content and recommendations.

We at the Revista Brasileira de Terapia Intensiva look forward to the next initiatives and guidelines from the *Diretrizes AMIB* project aimed to improve the quality of critical care in Brazil by delivering the best available care based on the best available evidence. Evidence-based practice brings pertinent, trustworthy information into clinical management and policy arenas by systematically acquiring, analyzing, and transferring research findings.^(3)^ Evaluating our documents based on the described aspects for quality assessment will ensure liability, scientific rigor, and the confidence of doctors, families, and health policy makers in our recommendations, as well as contribute to the rational use of resources to provide the best management available for critical care patients in Brazil.

## References

[1] Brouwers M, Kho ME, Browman GP, Cluzeau F, Feder G, Fervers B, Hanna S, Makarski J (2010). AGREE II: Advancing guideline development, reporting and
evaluation in healthcare. Can Med Assoc J.

[2] Schünemann HJ, Wiercioch W, Etxeandia I, Falavigna M, Santesso N, Mustafa R (2014). Guidelines 2.0: systematic development of a comprehensive
checklist for a successful guideline enterprise. CMAJ.

[3] Manchikanti L (2008). Evidence-based medicine, systematic reviews, and guidelines in
interventional pain management, part I: introduction and general
considerations. Pain Physician.

[4] Westphal GA, Garcia VD, Souza RL, Franke CA, Vieira KD, Birckholz VR (2016). Diretrizes para avaliação e validação
do potencial doador de órgãos em morte
encefálica. Rev Bras Ter Intensiva.

[5] GRADE Working Group (2008). GRADE: an emerging consensus on rating quality of evidence and
strength of recommendations. BMJ.

[6] Associação Médica Brasileira (AMB), Conselho Federal de Medicina (CFM) (2001). Projeto Diretrizes.

